# Generation of Skeletal Muscle Organoids from Human Pluripotent Stem Cells to Model Myogenesis and Muscle Regeneration

**DOI:** 10.3390/ijms23095108

**Published:** 2022-05-04

**Authors:** Min-Kyoung Shin, Jin Seok Bang, Jeoung Eun Lee, Hoang-Dai Tran, Genehong Park, Dong Ryul Lee, Junghyun Jo

**Affiliations:** 1 Department of Biomedical Science, College of Life Science, CHA University, 335 Pankyo-ro, Seongnam-si 13488, Korea; smksin0807@gmail.com (M.-K.S.); genehpark96@gmail.com (G.P.); 2 Stem Cell and Organoid Research Group, Okinawa Institute of Science and Technology Graduate University, 1919-1 Tancha, Onna-son, Kunigami-gun, Okinawa 904-0495, Japan; jinseok.bang2@oist.jp (J.S.B.); hoang.tran@oist.jp (H.-D.T.); 3 CHA Advanced Research Institute, CHA Bundang Medical Center, CHA University, 335 Pankyo-ro, Seongnam-si 13488, Korea; jel43@chamc.co.kr; 4 Department of Pharmacology, Ajou University School of Medicine, 164 Worldcup-ro, Yeongtong-gu, Suwon 16499, Korea; 5 Center for Convergence Research of Neurological Disorders, Ajou University School of Medicine, 164 Worldcup-ro, Yeongtong-gu, Suwon 16499, Korea; 6 Neuroscience Graduate Program, Department of Biomedical Sciences, Ajou University School of Medicine, 164 Worldcup-ro, Yeongtong-gu, Suwon 16499, Korea

**Keywords:** human pluripotent stem cells, skeletal muscle organoids, satellite cells, myogenesis, regeneration

## Abstract

In vitro organoids derived from human pluripotent stem cells (hPSCs) have been developed as essential tools to study the underlying mechanisms of human development and diseases owing to their structural and physiological similarity to corresponding organs. Despite recent advances, there are a few methodologies for three-dimensional (3D) skeletal muscle differentiation, which focus on the terminal differentiation into myofibers and investigate the potential of modeling neuromuscular disorders and muscular dystrophies. However, these methodologies cannot recapitulate the developmental processes and lack regenerative capacity. In this study, we developed a new method to differentiate hPSCs into a 3D human skeletal muscle organoid (hSkMO). This organoid model could recapitulate the myogenesis process and possesses regenerative capacities of sustainable satellite cells (SCs), which are adult muscle stem/progenitor cells capable of self-renewal and myogenic differentiation. Our 3D model demonstrated myogenesis through the sequential occurrence of multiple myogenic cell types from SCs to myocytes. Notably, we detected quiescent, non-dividing SCs throughout the hSkMO differentiation in long-term culture. They were activated and differentiated to reconstitute muscle tissue upon damage. Thus, hSkMOs can recapitulate human skeletal muscle development and regeneration and may provide a new model for studying human skeletal muscles and related diseases.

## 1. Introduction

The skeletal muscle is a post-mitotic tissue that constitutes up to 50% of human body mass. It is composed of contractile multinucleated myofibers generated by fusing differentiated muscle cells [1]. Skeletal muscle possesses an enormous regenerative ability to repair damaged tissue resulting from physiological conditions, such as exercise and aging, or diseases, such as cachexia, sarcopenia, and muscular dystrophies. A small population of myogenic stem/progenitor cells, known as satellite cells (SCs), play a role in the muscle fiber regenerative process [2,3,4]. Over the past decades, multiple attempts have been made to establish an appropriate model system to investigate embryonic and adult myogenesis regulated by the fate of SCs during muscle development and regeneration [5]. Despite recent progress, the necessity to develop novel in vitro experimental approaches to model skeletal muscle regeneration remains.

Recent advances in human pluripotent stem cell (hPSC)-based 3D in vitro organoid technology have provided new opportunities to understand human development and diseases associated with various organs, including the brain, spinal cord, liver, kidneys, and pancreas [6,7,8,9,10]. Despite its success in developing a wide range of human tissues, most differentiation protocols of hPSCs into skeletal muscle cells are solely based on two-dimensional (2D) methods. These conditions are unlikely to recapitulate the cytoarchitecture of developing skeletal muscle tissue with diverse myogenic cells, including SCs and myoblasts [11,12,13]. Several studies have reported the generation of 3D skeletal muscle tissues from hPSCs and modeled neuromuscular disorders and muscular dystrophies [14,15]. However, generating skeletal muscle-specific 3D tissue that can retain muscle regenerative capacities through damaged tissue repair remains a challenge.

Here, we introduce a novel method to recapitulate the features of embryonic myogenesis by paraxial mesodermal differentiation of hPSCs into human skeletal muscle organoids (hSkMOs), exhibiting structural organization of muscle cells and the different stages of heterogeneous myogenic cells. Notably, hSkMOs produce sustainable pair box 7-positive (PAX^+^) SCs in long-term cultures capable of generating muscle fibers contributing to muscle repair upon acute injury. Thus, hSkMOs may be valuable as an advanced muscle regeneration model and a viable platform for testing novel therapeutics.

## 2. Results

### 2.1. Generation and Characterization of hSkMOs from hPSCs

**Figure 1:**
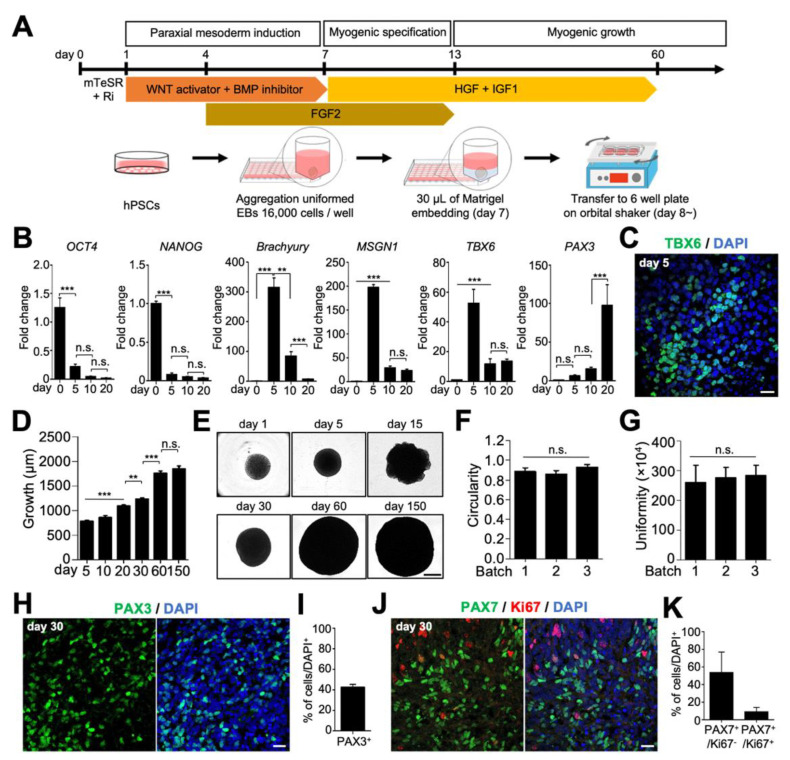
Generation and characterization of hSkMOs from hPSCs at early stages. (**A**) Schematic depicting the overall strategy to generate human skeletal muscle organoids (hSkMOs). (**B**) The gene expression of pluripotent (*OCT4*, *NANOG*), mesodermal posterior somitic (*Brachyury*, *MSGN1*, *TBX6*), and myogenic progenitor (*PAX3*) markers at different time points (days 0, 5, 10, and 20) of hSkMOs were identified by quantitative real-time reverse transcription-polymerase chain reaction (qRT-PCR) analysis. The qRT-PCR data were normalized to *GAPDH* expression. (**C**) Cryosection of the hSkMOs at day five stained for T-Box transcription factor 6 (TBX6). Scale bar, 20 μm. (**D**) The growth diameter demonstrates the average size (mean ± standard error of the mean (s.e.m.)) of hSkMOs from three batches. (**E**) The representative images of hSkMOs morphology are shown at each time point. Scale bar, 500 μm. (**F**) The circularity was calculated for each organoid in each batch using ImageJ software; mean ± s.e.m., *n* = 20. (**G**) Uniformity of hSkMOs was calculated for each organoid using ImageJ software; mean ± s.e.m., *n* = 20. (**H**) Cryosection of day 30 hSkMOs stained for paired box 3 (PAX3). Scale bar, 20 μm. (**I**) Quantification of the percentage of PAX3^+^ cells; mean ± s.e.m., *n* = 3. (**J**) Immunostaining of hSkMOs for PAX7 and Ki67 at day 30. Scale bar, 20 μm. (**K**) Quantification of the percentage of PAX7 ^+^ /Ki67^−^ and PAX7^+^/Ki67^+^; mean ± s.e.m., *n* = 3. Statistical analysis was performed using one-way analysis of variance (ANOVA), followed by Tukey’s multiple comparison test. ** *p* < 0.01, *** *p* < 0.001; n.s., not significant.

To generate hSkMOs, we developed a stepwise, pre-patterning differentiation protocol that guides hPSCs toward the paraxial mesodermal lineage, giving rise to myogenic progenitor cells and myoblasts (Figure 1A). First, hPSCs were dissociated into single cells and allowed to form uniformly sized (approximately 500 μm in diameter) embryoid bodies (EBs) in low-attachment V-shaped 96-well plates (Figure 1A). In our direct induction approach, we promoted paraxial mesodermal differentiation with WNT activation (CHIR99021), bone morphogenetic protein (BMP) inhibition (LDN193189), and fibroblast growth factor 2 (FGF2) signaling to generate presomitic cells in 3D culture conditions. Upon initial exposure to exogenous small molecules and growth factors, the time-course expression of stage-specific markers was profiled using quantitative reverse transcription-polymerase chain reaction (qRT-PCR) analysis on days 0, 5, 10, and 20. The expression of pluripotency markers octamer-binding transcription factor 4 (*OCT4*) and *NANOG* rapidly decreased, and the expression of *Brachyury* (mesoderm marker), T-Box transcription factor 6 (*TBX6*), and mesogenin 1 (*MSGN1*; presomitic markers) significantly increased within five days (Figure 1B). To further characterize paraxial mesodermal differentiation, we performed immunostaining on day five against TBX6, which demonstrated successful initial paraxial mesodermal commitment (Figure 1C).

After paraxial mesodermal induction for seven days, each organoid was embedded with growth factor-reduced Matrigel to provide an environment for enhancing 3D organization and then transferred to a six-well plate on an orbital shaker, similar to our previous organoid culture system (Figure 1A) [6,16]. The organoids were subjected to growth factor application to promote myogenic differentiation. Hepatocyte growth factor (HGF) and insulin-like growth factor 1 (IGF1) were added to the myogenic specification media, and hSkMOs were cultured until the day of analysis. The use of an orbital shaker significantly improved the viability, survival, and differentiation of hSkMOs increasing penetration rate of oxygen and nutrients into the core area of hSkMOs [17,18]. These myogenic organoids gradually grew to more than 1.5 mm in diameter by day 60 (Figure 1D,E). They appeared round-shaped, uniformly sized, and had relatively homogenous morphology; thus reducing batch-to-batch variability in size (Figure 1D–G). We reasoned that the myogenic progenitor markers, PAX3 and PAX7, would be expressed during myogenic specification because the qRT-PCR data demonstrated that the PAX3 expression gradually increased around day 20 (Figure 1B). We then performed cryosections to verify the hSkMO development on day 30. We observed that 43% of PAX3^+^ and 63% of PAX7^+^ cells were detected. Intriguingly, the myogenic cells appeared as clusters, and approximately 9% of PAX7^+^ cells were double-positive for Ki67 at day 30 (Figure 1H–K), demonstrating that proliferating cells are myogenic progenitors in hSkMOs. These data indicate that our in vitro hSkMO culture system recapitulates the features of embryonic skeletal muscle development through the differentiation of paraxial mesoderm into myogenic tissue with the expression of crucial marker genes.

### 2.2. Identifying and Characterizing Different Types of Skeletal Muscle Stem/Progenitor Cells during Myogenesis in hSkMOs

**Figure 2:**
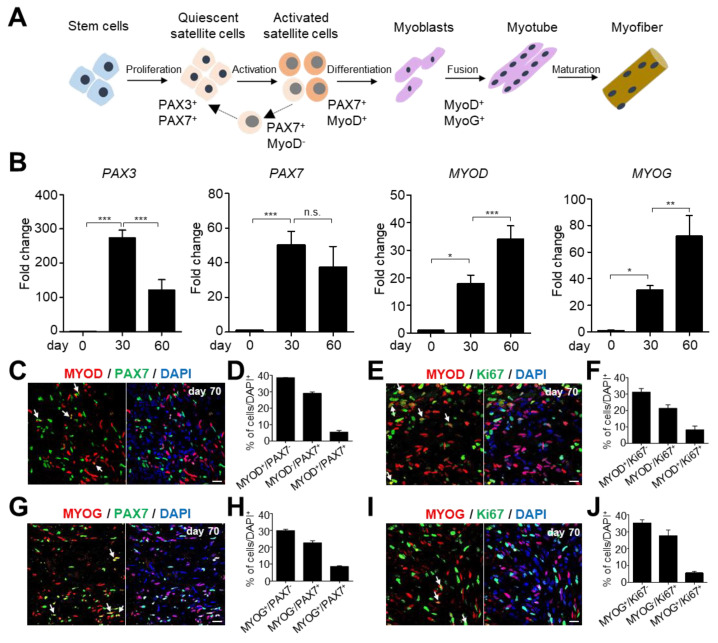
Characterization of SCs in hSkMOs. (**A**) Schematic representation of skeletal muscle differentiation and specific markers for each stage. (**B**) qRT-PCR data demonstrating the relative expression of SC (PAX3 and PAX7), activated SC (myoblast determination protein 1 (MYOD)), and myocyte (myogenin (MYOG)) markers at days 30 and 60 of hSkMOs. The qRT-PCR data were normalized to *GAPDH* expression. The bar graph represents the fold change in gene expression between the sample groups. Statistical analysis was performed using one-way ANOVA, followed by Tukey’s multiple comparison test. * *p* <0.05, ** *p* <0.01, *** *p* <0.001; n.s., not significant. (**C**) Cryosections of day 70 hSkMOs stained with specific antibodies against MYOD and PAX7. The quantifications are represented in (**D**); mean ± s.e.m., *n* = 3. (**E**) Cryosections of day 70 hSkMOs stained with specific antibodies against MYOD and Ki67. The quantifications are represented in (**F**) as mean ± s.e.m., *n* = 3. (**G**) Cryosections of day 70 hSkMOs stained with specific antibodies against MYOG and PAX7. The quantifications are represented in (**H**) as mean ± s.e.m., *n* = 3. (**I**) Cryosections of day 70 hSkMOs stained with specific antibodies against MYOG and Ki67. The quantifications are represented in (**J**); mean ± s.e.m., *n* = 3. Scale bars, 20 μm. White arrows indicate double-positive cells.

In both embryonic and adult myogenesis, myogenic stem/progenitor cells, such as SCs, are the major cell populations involved in skeletal muscle differentiation and regeneration [5]. SCs reside in a specific muscle stem cell niche on the muscle fiber and remain quiescent until muscle damage-induced activation during acute injury or environmental conditions [2]. Transcription factors from the PAX gene family, PAX3 and PAX7, are crucial for myogenesis and SCs determination. Additionally, muscle regulatory transcription factors (MRFs) such as myoblast determination protein 1 (MYOD) and myogenin (MYOG) are critical for myogenic progenitor cell determination and terminal differentiation (Figure 2A) [19]. To check the expression of cell type-specific markers, such as skeletal muscle stem/progenitor and differentiating cells, we performed qRT-PCR analysis on hSkMOs on days 30 and 60. PAX3 and PAX7 (SC markers) expression peaked on day 30 (Figure 2B). The expression of MYOD (proliferating and activated SC marker) and MYOG (differentiated myocyte marker) increased over time (Figure 2B). These data demonstrate that PAX3- and PAX7-expressing muscle stem/progenitor cells are the major population during the early stage of myogenesis and remain as resident SCs in mature skeletal muscle tissues. Activated muscle progenitor cells and differentiated myoblasts are augmented over time.

To further characterize and quantify the specific myogenic populations within the hSkMOs, we conducted immunohistochemistry analysis and observed distinct cell type-specific marker expression in hSkMOs at day 70. MYOD^−^/PAX7^+^, MYOD^+^/PAX7^+^, and MYOD^+^/Ki67^+^ cells accounted for 29%, 6%, and 8% of the putative quiescent, activated, and proliferating SCs, respectively. MYOD^+^/PAX7^−^ cells constituted 39% of differentiating myoblasts (Figure 2C–F). Additionally, MYOG^−^/PAX7^+^ cells constituted 23% of putative quiescent SCs, and MYOG^+^/PAX7^−^ cells accounted for 30% of differentiated myocytes (Figure 2G–J). Intriguingly, we observed that 8% and 6% of the MYOG^+^ cells in hSkMOs co-expressed PAX7 and Ki67, respectively (Figure 2G–J). Collectively, these data demonstrate that our hSkMOs recapitulated myogenesis progression and produced myogenic stem/progenitor and differentiated cells during long-term culture.

### 2.3. Maturation of Skeletal Muscle Cells in hSkMOs

**Figure 3:**
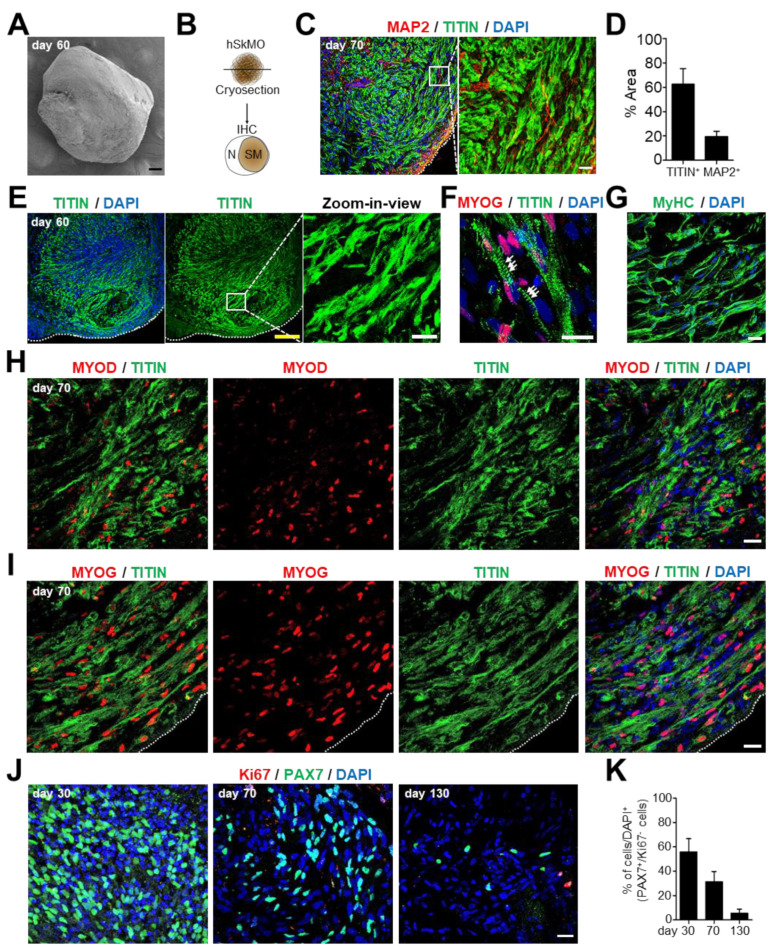
Characterization of mature hSkMOs. (**A**) SEM image of day 60 hSkMO. Scale bar, 100 μm. (**B**) Schematic representation of cryosection and the predicted proportion of skeletal muscle (SM) and neuron (N) region in hSkMOs. (**C**) Immunofluorescence analysis of hSkMOs cryosection at day 70 confirmed the presence of TITIN^+^ and MAP2^+^ cells. Scale bar, 20 μm. (**D**) The percentage of TITIN area at day 60 of hSkMOs was calculated using ImageJ software. (**E**) Immunostaining of hSkMOs at day 60 with sarcomeric protein TITIN antibody and zoom-in view to illustrate striated muscle by TITIN antibody staining. Yellow scale bar, 200 μm. White scale bar, 20 μm. (**F**) Immunostaining of TITIN and MYOG antibodies on hSkMOs. Sarcomeric bands are marked with a white arrow. Scale bar, 10 μm. (**G**) Cryosection of day 60 hSkMO stained with specific antibody against MyHC. Scale bar, 20 μm. (**H**,**I**) Day 70 hSkMOs were stained with MYOD, MYOG, and TITIN. Scale bar, 20 μm. (**J**) Immunostaining of hSkMOs at days 30, 70, and 130 demonstrating the presence of SCs using PAX7 and Ki67 antibodies. Scale bar, 20 μm. (**K**) The percentage of PAX7^+^/Ki67^−^ cells were calculated using ImageJ software; mean ± s.e.m., *n* = 3.

hSkMOs grew exponentially in size within 2 months, presumably by increasing myogenic stem/progenitor and mature muscle cells, and the organoid growth rate steadily diminished in size from 2 months (Figure 1D). This result demonstrated that embryonic myogenesis for the development of skeletal muscle tissue was complete and that hSkMOs consisted of a large population of terminally differentiated myogenic cells and a small population of preserved myogenic stem/progenitor cells (Figure 2C–J). We performed scanning electron microscopy (SEM) imaging (Figure 3A) to further examine the cytoarchitecture of hSkMOs after 2 months. Confocal microscopic images revealed the presence of abundant myofiber-like structures (Figure 3B–F). We speculated that the hSkMOs should contain paraxial mesodermal progenitor-derived myogenic cells and neuromesodermal progenitor-derived neurons following early embryonic mesodermal development. Thus, we performed immunohistochemical analysis to evaluate the proportion of myogenic and neuronal cells (Figure 3C,D). We confirmed that hSkMOs consisted of a substantial proportion of TITIN^+^ muscle cells, and microtubule-associated protein 2 (MAP2)-positive neurons were observed within a particular portion of hSkMOs (Figure 3C). To quantify the area of skeletal muscle tissue within these hSkMOs, we measured the fluorescence coverage of TITIN and MAP2 from confocal microscopy images. The results indicated that 62% of the TITIN^+^ skeletal muscle region and 20% of the MAP2^+^ neuronal region were distinctively separated (Figure 3D). Furthermore, we analyzed the expression levels of fibro-adipogenic progenitors (FAPS) markers, platelet-derived growth factor receptor A (*PDGFRα*), and PR domain containing 16 (*PRDM16*) using qRT-PCR analysis. The expression levels of PDGFR and PRDM16 were drastically increased on day 60 (Supplementary Figure S1).

Skeletal muscles are composed of mature myofibers containing numerous myofibrils, and sarcomeres are the basic functional units of skeletal muscles [20]. The expression of sarcomeric proteins and the organization of sarcomeric structures should indicate myofiber formation and maturation of skeletal muscle tissue. To define muscle maturation during the long-term culture of the hSkMOs, we performed immunohistochemistry analysis. The results revealed that highly organized skeletal muscle exhibited numerous sarcomeric bands labeled by TITIN antibody (Figure 3E,F) as well as myotube maturation marker by myosin heavy chain (MyHC) antibody (Figure 3G). Moreover, we observed that MYOD^+^ and MYOG^+^ myogenic cells coexisted within TITIN^+^ myofibers (Figure 3H,I). To further characterize the presence of sustainable SCs within mature hSkMOs, we quantified the amount of dormant SCs by confocal microscopy imaging. The data revealed that approximately 56%, 31%, and 5% of PAX7^+^/Ki67^−^ putative dormant SCs existed throughout differentiation of hSkMOs at days 30, 70, and 130, respectively (Figure 3J,K). Hence, these data demonstrate that structurally well-developed myofibers within hSkMOs expressing sarcomeric protein and terminally differentiated myocyte markers demonstrate mature skeletal muscle properties. Additionally, dormant PAX7^+^ SCs were sustained in hSkMOs over time, indicating the regenerative potential of our in vitro 3D skeletal muscle tissue.

### 2.4. Toxin-Induced Muscular Degeneration and Regeneration Capacity of hSkMOs

**Figure 4:**
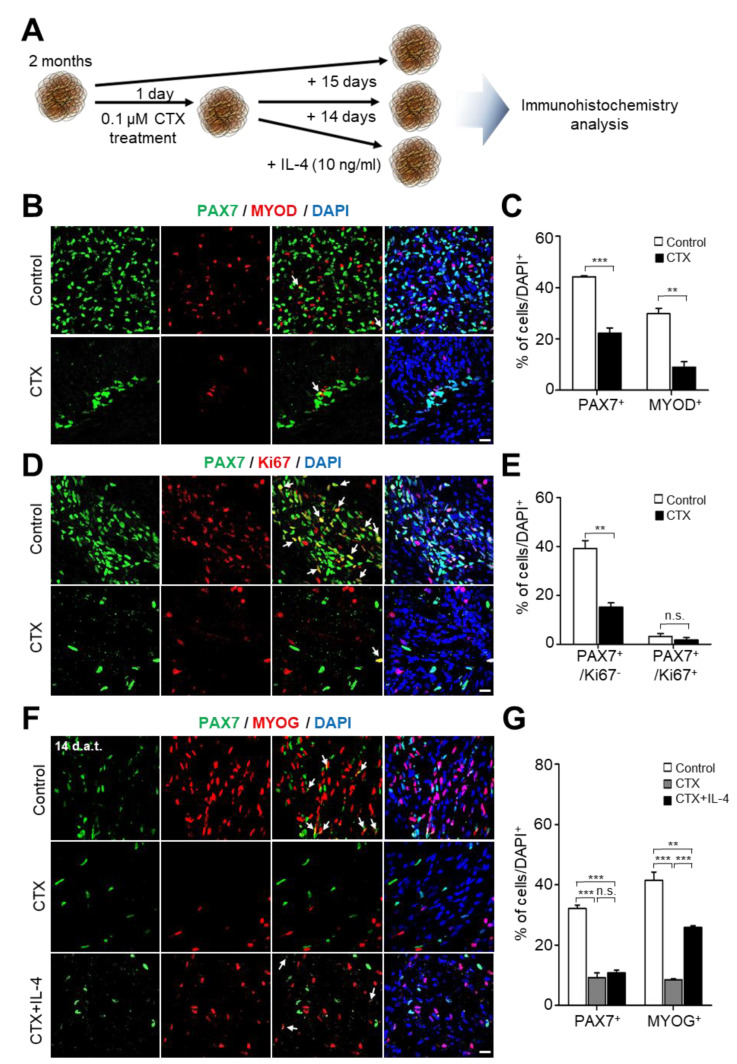
Characterization of mature hSkMOs. (**A**) Schematic diagrams for skeletal muscle regeneration model using hSkMOs. (**B**) Cryosections of hSkMOs stained with specific antibodies against MYOD and PAX7. Scale bars, 20 μm. The quantifications are shown in (**C**); mean ± s.e.m., *n* = 3; unpaired *t*-test. Statistics: ** *p* <0.01, *** *p* <0.001. (**D**) Cryosections of day 50 hSkMOs stained with specific antibodies against Ki67 and PAX7. Scale bars, 20 μm. The quantifications are shown in (**E**); mean ± s.e.m., *n* = 3; unpaired *t*-test. Statistics: **p <0.01; n.s., not significant. (**F**) Cryosection of hSkMOs stained with specific antibodies against MYOG and PAX7. Scale bar, 20 μm. The quantifications are shown in (**G**); mean ± s.e.m., *n* = 3. Statistical analysis was performed using one-way ANOVA, followed by Tukey’s multiple comparison test. Statistics: ** *p* < 0.01, *** *p* < 0.001; n.s., not significant. White arrows indicate double-positive cells.

Given the presence of sustainable SCs in mature hSkMOs (Figure 3J,K), we expected hSkMOs to have regenerative potential by activating quiescent SCs and subsequent differentiation into myocytes upon muscle damage. The cardiotoxin (CTX) injury model, which induces myolysis, has been widely used to induce skeletal muscle damage in mouse and cellular models [21,22]. Treatment with CTX in culture media induces muscle inflammation and myofiber degeneration followed by a regenerative response. The damaged myofibers are typically restored in 2–3 weeks after CTX-induced damage [21]. With this notion, we examined muscle damage in hSkMOs by treatment with CTX and the intrinsic regenerative ability of SCs (Figure 4A). Adding CTX (0.1 μM) in the culture media induced acute muscle damage in hSkMOs. We observed a drastic reduction in PAX7^+^ and MYOD^+^ cells in hSkMOs through immunohistochemistry analysis (Figure 4B,C). Although non-dividing quiescent SCs (PAX7^+^/Ki67^−^) were reduced by more than half in the CTX treatment group compared to the control group, PAX7^+^/Ki67^+^ cells were retained (Figure 4D,E).

Several studies have shown that other cell types, such as mesoangioblasts, vessel-associated muscle progenitors, fibroadipogenic progenitors, and macrophages, contribute to myogenesis in the SC niche [23,24]. Given the limitations of cell-type compositions for non-myogenic cells and to accelerate muscle regeneration in our hSkMOs induced by CTX treatment, we supplemented cytokines released from the other cells in the SC niche upon muscle damage. Interleukin-4 (IL-4) is secreted from non-myogenic interstitial stem cells present in adult muscle and improves skeletal muscle regeneration and maturation of myotubes [23,25]. IL-4 was added to the medium to promote muscle regeneration in CTX-injured hSkMOs for 14 days to evaluate the extent of muscle regeneration. We then immunostained the hSkMOs using antibodies against PAX7 and MYOG to check for muscle regeneration of augmented SCs and myocytes. The CTX-untreated control hSkMOs showed a considerable presence of PAX7^+^ SCs (32%) and MYOG^+^ myocytes (41%) (Figure 4F,G). Intriguingly, we observed a significant increase in MYOG^+^ myocytes (26%) in the CTX-injured hSkMOs with the treatment of IL-4 compared to that in MYOG+ myocytes (9%) in CTX-injured hSkMOs (Figure 4F,G). Collectively, these data indicate that hSkMOs exhibited regenerative potential upon muscle damage and were able to mimic adult myogenesis promoted by cytokine treatment.

## 3. Discussion

Over the past decades, various animal models of muscle degeneration, including cytotoxin-induced injury, crush injury, and transgenic mice carrying muscular dystrophy-associated genetic mutations, have been utilized as standard muscle regeneration models for basic research. Animal models have been invaluable for studying the mechanisms of muscle regeneration regulated by SCs. However, they do not faithfully recapitulate a diverse range of disease phenotypes, and the lack of human-like pathology limits their application in preclinical research [26]. In this context, we suggested the establishment of reliable human muscle cell-based in vitro modeling systems.

In this study, we demonstrated that hPSCs could be committed to the paraxial mesodermal lineage and further differentiate into 3D hSkMOs comprising sustainable SCs and distinct myofibers with sarcomere protein expression and structural organization. Most approaches to skeletal muscle differentiation have been developed based on 2D culture systems to differentiate hPSCs into myofibers [11,12,13]. Although 2D culture systems are straightforward and valuable for in vitro modeling studies, the lack of a native microenvironment and SC niche restricts their application to model adult myogenesis and muscle regeneration [27].

In our protocol, we utilized WNT activator and BMP inhibitors at the beginning of the EB differentiation to improve paraxial mesodermal induction, combined with stimulation of FGF2 signaling upon Matrigel embedding to promote the 3D structural organization. HGF and IGF1 were later added to accelerate myogenic specification and further myofiber differentiation. Particularly, the myogenic tissues were poorly developed when hSkMOs were embedded into Matrigel either at the beginning of EB differentiation or in the later stages. Thus, we optimized the specific time for Matrigel embedding at day seven (Figure 1A). We observed neural cells presumably derived from neuromesodermal progenitors. FGF2 was withdrawn to arrest neural lineage to achieve exclusive muscle tissue development, and HGF and IGF1 treatment were prolonged to propagate myogenic progenitors [28]. Remarkably, based on immunohistochemical analysis, we found that 62% of the proportion was skeletal muscle tissue based on measuring the TITIN^+^ striated muscle tissue area (Figure 3D). Within the skeletal muscle area in hSkMOs, we observed about 31–35% of PAX7^+^ myogenic stem/progenitor cells, 40–44% of MYOD^+^ activated/committed myoblasts, and 38–41% of MYOG^+^ myocytes (Figure 2C–J). Notably, 31% of PAX7^+^/Ki67^−^ and 29% of MYOD^−^/PAX7^+^ non-dividing quiescent SCs were found in mature hSkMOs (Figure 2 and Figure 3). These features indicated that our hSkMOs could effectively recapitulate embryonic myogenesis and possess regenerative potential regulated by sustainable SCs. Using comprehensive immunohistochemical analysis at multiple stages, we demonstrated the presence of different myogenic cells. However, future studies using a single-cell RNA sequencing approach may be required to further characterize different types of cells existing in hSkMOs by cell type-specific comparative transcriptomic analysis [14].

In homeostatic skeletal muscles, SCs generally remain in a quiescent state [5]. In response to muscle injuries, SCs are activated and promote myogenic regenerative activity to repair damaged tissues [29,30]. Other cell types can contribute to myogenesis, and SCs are affected by non-myogenic cells that secrete several cytokines such as IL-4 and IL-13 [31,32]. In particular, IL-4 can influence the inflammatory system, promote an anti-inflammatory muscle microenvironment, and contribute to SCs differentiation; thus promoting muscle regeneration [2]. Although the de novo organoids generated from hPSCs have tremendous potential in the study of organ development and pathogenesis, they do not fully recapitulate the in vivo native microenvironment. This includes vasculature formation and cellular composition, including immune and non-myogenic cells. Upon muscle damage, various cells in the SC niche orchestrate myogenesis and promote muscle regeneration through interactions with their niche. As these hSkMOs are devoid of immune cells such as macrophages, treatment with extrinsic cytokines in the culture media was necessary to promote muscle regeneration. They demonstrated accelerated regeneration of damaged muscle tissue. The most interesting aspect of our hSkMOs is their remarkable potential to foster in vitro model systems by co-culture with other cell types or tissues and investigate their interactions. Additionally, hSkMOs will be invaluable for evaluating the efficacy of therapeutic candidates, such as small molecules, growth factors, and cytokines.

In conclusion, we successfully established a new method to generate hSkMOs that recapitulate embryonic myogenesis and demonstrate their muscle regeneration potential. Our hSkMOs could serve as a versatile platform to study aspects of human muscle biology with environmental factors and intrinsic modulation in 3D culture conditions because of the accessibility of monitoring the properties of muscle tissue. Additionally, hSkMO could provide a more feasible in vitro model system to investigate the molecular and cellular mechanisms of muscle regeneration and identify novel therapeutic candidates. hSkMOs would allow us to perturb multiple pathways by CRISPR/Cas9-mediated genetic mutations and/or chemical insults to recapitulate muscle degeneration and progressive pathological features of muscle-wasting disorders. This would facilitate further disease modeling studies and large-scale drug tests/screens for a broad array of skeletal muscular disorders, including Duchenne muscular dystrophy and sarcopenia.

## 4. Materials and Methods

### 4.1. Culture of Human Pluripotent Stem Cells (hPSCs)

Two different hPSCs were used in this study: H9 (WA09; passage 35) was purchased from the WiCell Research Institute (Madison, WI, USA) and CHA-SCNT-PSC-18 (Korea Stem Cell Registry code hES12019001), which we previously established [33,34]. H9 cells were maintained in the mTeSR medium (Stem Cell Technologies, BC, Canada). CHA-SCNT-PSC-18 was cultured on mitotically inactivated mouse embryonic fibroblasts (CF1; Jackson Laboratory, CA, USA) in Dulbecco’s modified Eagle medium (DMEM)/F12 (Nacalai Tesque, Kyoto, Japan) supplemented with 20% knockout serum replacement (KSR; Thermo Fisher Scientific), 0.1 mM beta-mercaptoethanol (Thermo Fisher Scientific, MA, US), 1% non-essential amino acids (NEAA; Thermo Fisher Scientific), and 4 ng/mL recombinant human FGF2 (Gibco). The hPSCs medium was changed daily, and cells were passaged every 4–5 days. The hPSCs were maintained on Matrigel (BD Biosciences, San Jose, CA, USA)-coated plates in mTeSR and passaged when reaching 70–80% confluency. Cells before passage 45 were used to generate hSkMOs.

### 4.2. Generation of Human Skeletal Muscle Organoids

For the generation of hSkMOs, hPSCs were dissociated into single cells using TrypLE solution (Thermo Fisher Scientific, Waltham, MA, US) and seeded at 1.6 × 10^4^ cells per well in a low-attachment V-shaped 96-well plate (Sumilon, Tokyo, Japan; MS-9096V) in mTeSR supplemented with 10 μM Rho kinase (ROCK) inhibitor (Tocris, Avon, UK) to form EBs on day zero. After 24 h, the EBs were cultured in a paraxial mesoderm induction medium consisting of DMEM/F12, insulin-transferrin-selenium (ITS; Life Technologies, Carlsbad, CA, USA), 1% non-essential amino acid (NEAA), 1% penicillin/streptomycin containing 3 μM CHIR99021 (Stem Cell Technologies, Vancouver, BC, Canada), and 0.5 μM LDN193189 (Stemgent, San Diego, CA, USA) for 3 days. On day four, 20 ng/mL FGF2 (Gibco) was added for 9 days to promote the posterior mesoderm development. On day seven, organoids were embedded in growth factor-reduced Matrigel and cultured in a myogenic specification medium consisting of DMEM/F12, 15% KSR, 1% NEAA, 1% penicillin/streptomycin, 0.1 mM 2-mercaptoethanol containing 10 ng/mL HGF (Peprotech, Rocky Hill, NJ, USA), 2 ng/mL IGF1 (Sigma-Aldrich, Saint Louis, MO, USA), and 20 ng/mL FGF2. After 24 h, the embedded organoids were transferred to an ultra-low attachment six-well plate (Corning Costar) and incubated with 4 mL of myogenic specification medium on an orbital shaker (Stuart) rotating at 90 rpm. On day 13, organoids were maintained in a myogenic specification medium without FGF2. The detailed procedure for the generation of hSkMOs is depicted in Figure 1A.

### 4.3. Immunofluorescence Analyses Using Cryosection

For immunofluorescence analyses, hSkMOs were washed with phosphate-buffered saline (PBS) twice and then fixed with 4% paraformaldehyde (PFA) at 4 °C overnight. Fixed hSkMOs were washed with PBS then placed in PBS containing 30% sucrose solution until hSkMOs were dispersed in solution. Subsequently, hSkMOs were cryo-embedded in optimal cutting temperature (OCT) compound (Sakura Tissues Tek) and sectioned at a thickness of 16 μm using a cryostat (Leica CM1950). Cryosectioned slices were washed with PBS before permeabilization with 0.5% Triton X-100 in PBS for 1 h. The slices were blocked with a blocking buffer consisting of 10% donkey serum in PBS with 0.3% Triton X-100 and 1% bovine serum albumin (BSA) for 1 h. Primary antibodies were diluted in antibody buffer consisting of 2% donkey serum in PBS with 0.1% Triton X-100 at 4 °C overnight. The primary antibodies and their dilutions are listed in Table 1. On day two, the slices were washed with PBS containing 0.1 % Tween-20 twice every 5 min. Secondary antibodies diluted in antibody buffer were applied to the sections for 1 h at room temperature. After 1 h, the sections were washed twice for 5 min each before mounting. Alexa Fluor 488 and 594-conjugated donkey antibodies (Invitrogen) were used at 1:1000 dilution. Images were captured using a confocal microscope (Zeiss LSM 880) in Airyscan mode. Sample images were prepared using ImageJ software (NIH).

### 4.4. RNA Extraction, Brachyury Reverse Transcription, Quantitative Real-Time RT-PCR Analysis

Total RNA was isolated from the hSkMOs using RNAiso Plus containing 38% phenol (#9109; Takara Bio Inc., Shiga, Japan). Then, 1 μg of RNA was reverse transcribed into first-strand cDNA using the TOPscript™ RT DryMIX (dT18 plus) (Enzynomics Inc., Daejeon, Korea) and T100™ Thermal Cycler (Bio-Rad, Hercules, CA, USA) according to the manufacturer’s instructions. For qRT-PCR analysis, amplification reactions were performed using SYBR Green TOPreal ™ qPCR 2X PreMIX (Enzynomics) on a CFX96™ Real-Time System, C1000™ Touch Thermal Cycler (Bio-Rad) according to the manufacturer’s protocol. The primer sequences used for qRT-PCR are listed in Table 2.

### 4.5. Scanning Electron Microscopy (SEM)

For electron microscopy analysis, hSkMOs were fixed in glutaraldehyde (2.5%, *v*/*v*) in phosphate buffer (0.1 M; pH7.4) for 30 min and washed thrice every 10 min using 0.1 M phosphate buffer. After primary fixation, specimens were post-fixed in osmium tetroxide (1%, *w*/*v*) in phosphate buffer (0.1 M; pH7.4) for 30 min and rinsed with deionized water thrice every 10 min. Subsequently, fixed specimens were dehydrated with a series of graded ethanol from 70 to 100% for 10 min each. After dehydration, the specimens were dried at −20 °C overnight and then coated with gold (10 nm thick) for 2 min. The sputter-coated specimens were visualized for imaging analysis using an SEM (JSM7900F, JEOL, Tokyo, Japan) equipped with a cold cathode emitter set at an acceleration voltage of 5.0 kV.

### 4.6. Muscle Injury Procedures and Regeneration

The hSkMOs on day 50 were treated with 0.1 μM cardiotoxin (CTX) for 24 h at 37 °C. For the regeneration model, hSkMOs injured by CTX were incubated with or without IL-4 (10 ng/mL) for 14 days. All samples were subjected to immunofluorescence analysis. The detailed procedure for the muscle regeneration model of hSkMOs is shown in Figure 4A.

### 4.7. Quantification and Statistical Analyses

Statistical analyses were performed using GraphPad Prism software V5. “*n*” indicates the number of independent experiments, which were conducted at least in triplicate. The results are expressed as mean ± standard error (exact *n* numbers are described in the figure legends). Statistical analyses were followed by an unpaired Student’s *t*-test when comparing two samples and by one-way ANOVA with Tukey’s multiple comparison test when comparing more than two samples.

## Figures and Tables

**Table 1:** Antibodies for immunofluorescence analyses.

Primary Antibody Name	Catalogue Number	Company	Host Species	Dilution
Ki67	550609	BD	Mouse	1:200
Ki67	ab15580	Abcam	Rabbit	1:200
PAX3	PAX3	DSHB	Mouse	1:100
PAX7	PAX7	DSHB	Mouse	1:200
MAP2	ab5392	Abcam	Chicken	1:2000
MyHC	5-6-s	DSHB	Mouse	1:25
MYOD	ab133627	Abcam	Rabbit	1:500
MYOG	ab124800	Abcam	Rabbit	1:500
TBX6	AF4744	R&D Systems	Goat	1:50
TITIN	9D10	DSHB	Mouse	1:200

**Table 2:** Primers used for qRT-PCR experiments.

Gene	Forward Primer	Reverse Primer
*GAPDH*	CAAGATCATCAGCAATGCCTCCTG	GCCTGCTTCACCACCTTCTTGA
*OCT4*	GTGGAGGAAGCTGACAACAA	ATTCTCCAGGTTGCCTCTCA
*NANOG*	TTTGTGGGCCTGAAGAAAACT	AGGGCTGTCCTGAATAAGCAG
*Brachyury*	TTCATAGCGGTGACTGCTTATCA	CACCCCCATTGGGAGTACC
*MSGN1*	CTGCACACCCTCCGGAATT	CTCTGCCGCGGTTAAGGAG
*TBX6*	CATCCACGAGAATTGTACCCG	AGCAATCCAGTTTAGGGGTGT
*PAX3*	AGCTCGGCGGTGTTTTTATCA	CTGCACAGGATCTTGGAGACG
*PAX7*	CGTGCTCAGAATCAAGTTCG	GTCAGGTTCCGACTCCACAT
*MYOD*	ACTTTCTGGAGCCCTCCTGGCA	TTTGTTGCACTACACAGCATG
*MYOG*	GCCAACCCAGGGGATCAT	CCCGGCTTGGAAGACAATCT

## Data Availability

Not applicable.

## Supplementary Materials

The following supporting information can be downloaded at https://www.mdpi.com/article/10.3390/ijms23095108/s1.
