# Supplementary material for: Generation of Skeletal Muscle Organoids from Human Pluripotent Stem Cells to Model Myogenesis and Muscle Regeneration
Source: Int J Mol Sci. 2022 May 4;23(9):5108. doi: 10.3390/ijms23095108 (PMC9103168; doi:10.3390/ijms23095108)
Supplement: Supplementary file 1 [file ijms-23-05108-s001.zip › ijms-1691083-supplementary.pdf]

## Supplementary Materials

# Generation of Skeletal Muscle Organoids from Human Pluripotent Stem Cells to Model Myogenesis and Muscle Regeneration

**Min-Kyoung Shin, Jin Seok Bang, Jeoung Eun Lee, Hoang-Dai Tran, Genehong Park, Dong Ryul Lee and Junghyun Jo**

1) **Supplementary Figure and Figure Legends**
2) **Supplementary Table**

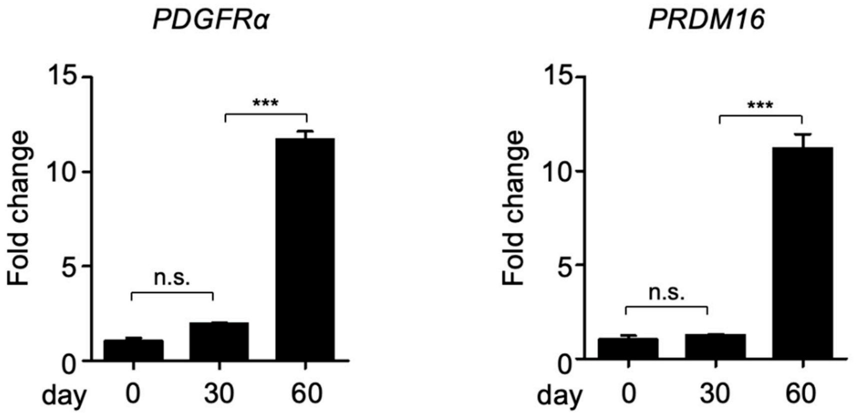


**Supplementary Figure S1. Relative expression levels of the FAPS target gene in hSkMOs.** The gene expression of FAPS makers (*PDGFRα* and *PRDM16*) at different time points (day 0, 30, and 60) of hSkMOs were identified by qRT-PCR analysis. The qRT-PCR data were normalized to *GAPDH* expression. Statistical analysis was performed using one-way ANOVA, followed by Tukey's multiple comparison test. ***$p$ <0.001; n.s., not significant.

**Supplementary Table S1. Primers used for qRT-PCR analysis.**

| Gene | Forward Primer | Reverse Primer |
|---|---|---|
| *PDGFRα* | TGGCAGTACCCCATGTCTGAA | CCAAGACCGTCACAAAAAGGC |
| *PRDM16* | GTTCTGCGTGGATGCAAATCA | GGTGAGGTTCTGGTCATCGC |
